# TDP-43 aggregation mirrors TDP-43 knockdown, affecting the expression levels of a common set of proteins

**DOI:** 10.1038/srep33996

**Published:** 2016-09-26

**Authors:** S. Prpar Mihevc, Marco Baralle, Emanuele Buratti, Boris Rogelj

**Affiliations:** 1 Department of Biotechnology, Jožef Stefan Institute, Jamova 39, SI-1000 Ljubljana, Slovenia; 2 International Centre for Genetic Engineering and Biotechnology (ICGEB), Padriciano 99, IT-34149 Trieste, Italy; 3 Biomedical Research Institute (BRIS), Puhova 10, SI-1000 Ljubljana, Slovenia; 4 Faculty of Chemistry and Chemical Technology, University of Ljubljana, Večna pot 113, SI-1000 Ljubljana, Slovenia

## Abstract

TDP-43 protein plays an important role in regulating transcriptional repression, RNA metabolism, and splicing. Typically it shuttles between the nucleus and the cytoplasm to perform its functions, while abnormal cytoplasmic aggregation of TDP-43 has been associated with neurodegenerative diseases amyotrophic lateral sclerosis (ALS) and frontotemporal lobar degeneration (FTLD). For the purpose of this study we selected a set of proteins that were misregulated following silencing of TDP-43 and analysed their expression in a TDP-43-aggregation model cell line HEK293 Flp-in Flag-TDP-43-12x-Q/N F4L. Following TDP-43 sequestration in insoluble aggregates, we observed higher nuclear levels of EIF4A3, and POLDIP3β, whereas nuclear levels of DNMT3A, HNRNPA3, PABPC1 and POLDIP3α dropped, and cytoplasmic levels of RANBP1 dropped. In addition, immunofluorescence signal intensity quantifications showed increased nuclear expression of HNRNPL and YARS, and downregulation of cytoplasmic DPCD. Furthermore, cytoplasmic levels of predominantly nuclear protein ALYREF increased. In conclusion, by identifying a common set of proteins that are differentially expressed in a similar manner in these two different conditions, we show that TDP-43 aggregation has a comparable effect to TDP-43 knockdown.

TDP-43 protein, encoded by the *TARDBP* gene, plays an important role in regulation of several processes, including microRNA processing, apoptosis, cell division, transcription, translation, splicing, axonal transport, and neurite outgrowth^1^,^2^. Its major distinguishing features are the ability to bind RNA in a very specific manner through two RNA recognition motifs (RRM) and the C-terminal portion of the protein, which includes a glycine-rich domain that is involved in most of the protein interactions described^3^. This region contains a glutamine/asparagine (Q/N) prion-like domain that participates in protein–protein interactions and in the TDP-43 aggregation process^4^,^5^. Typically, TDP-43 is shuttled between the nucleus and the cytoplasm to perform its functions^6^,^7^,^8^. Depletion of TDP-43 is embryonic lethal at very early stages of development and its overexpression above normal levels is highly toxic to cells, especially neurons^9^,^10^,^11^.

Abnormal cytoplasmic and occasional intranuclear aggregation of TDP-43 has been associated with amyotrophic lateral sclerosis (ALS) and frontotemporal lobar degeneration (FTLD-TDP)^12^,^13^. The discovery of missense mutations of *TARDBP* in familial and sporadic ALS cases proved the essential role of abnormal TDP-43 in disease^14^. Wild-type TDP-43 itself is intrinsically aggregation-prone as well as toxic but a few ALS-causing mutations appear to significantly exaggerate the aggregation process^15^,^16^. From the point of view of the pathology, however, it is important to highlight that wild-type cytoplasmic TDP-43 positive inclusions can be found in 95% of all ALS and 60% of FTLD cases, which are now termed TDP-43 proteinopathies^12^,^17^,^18^. TDP-43 positive cytoplasmic inclusions have also been described in 57% of Alzheimer’s disease cases, 20% of Dementia with Lewy Bodies, Pick’s disease, hippocampal sclerosis, corticobasal degeneration, Huntington disease, Parkinson’s disease, argyrophilic grain disease, and in a variety of other neurodegenerative conditions^19^,^20^. The histology in all these cases is similar, with TDP-43 present in cytoplasmic inclusions in glia and neurons, thus partially or totally cleared from the nucleus^21^,^22^. Taken together, aggregation of TDP-43 is most probably the root cause of ALS/FTLD either through a gain of toxic function (GOF) on its own or through a loss of function (LOF) with sequestration and depletion of nuclear TDP-43^23^,^24^ or both^25^. It is therefore of prime importance to better characterize its consequences at the cellular level. In this respect, previous studies demonstrated the effect of TDP-43 knockdown on the transcriptome^2^,^26^ and recently on proteome of SH-SY5Y cells^27^ and cytotoxicity has been observed to increase following cytoplasmic internalisation of TDP-43 containing inclusions bodies^28^. In general, aggregation-prone proteins that have been targeted to cytoplasm, show that cytoplasmic aggregates interfere with nuclear protein transport and inhibit mRNA transport^29^.

In this study, however, we have specifically investigated whether the aggregation/sequestration of TDP-43 correlated with its loss of function by comparing the expression changes of selected proteins responsive to silencing of TDP-43 in an aggregation and sequestration model cell line HEK293 Flp-in Flag-TDP-43-12x-Q/N F4L. Our results show that differential expression of proteins in TDP-12xQ/N-F4L cells correlated with proteomic results of TDP-43 knockdown in SH-SY5Y, revealing a common set of proteins whose expression is influenced via TDP-43 aggregation or knockdown.

## Results

For the purpose of this study, we selected 13 proteins (Table 1) whose expression levels we previously demonstrated to be affected by TDP-43 knockdown^27^. These particular proteins where selected based on their function, association with ALS and FTLD-TDP, and availability of the antibodies. We analyzed their expression in HEK293 Flp-in Flag-TDP-43-12x-Q/N F4L cell line, comparing protein expressions before and after induction of Flag-TDP-43-12x-Q/N expression. We chose the mutant overexpressing Flag-TDP-43-12x-Q/N F4L, with double-site mutations F147/149L and F229/231 in RRM1 and RRM2, due to its inability to bind RNA and thus downregulate the levels of soluble endogenous TDP-43 through the negative feedback loop^6^,^30^. In this manner, we sought to replicate the most likely disease-condition where aggregates form in the presence of fully functional endogenous *TARDBP* gene expression.

**Figure 1:**
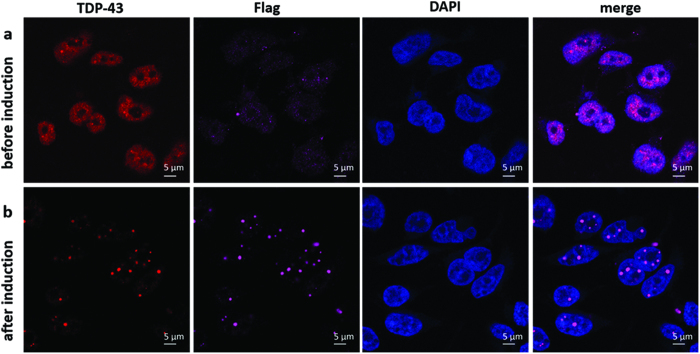
Cellular localisation of TDP-43 before (**a**) and after (**b**) induction of expression of Flag-TDP-43-12xQ/N F4L. Scale bars are 5 μm.

**Figure 2:**
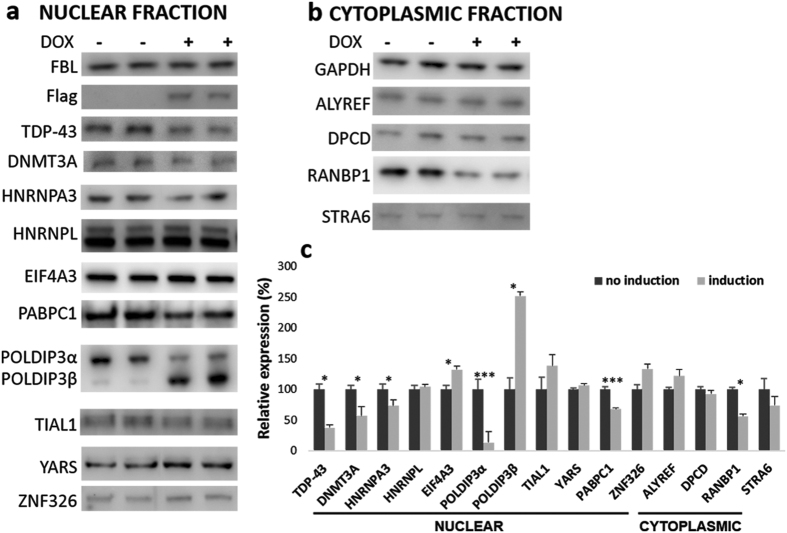
Expression of selected proteins in nuclear (**a**) or cytoplasmic (**b**) fractions of HEK Flp-in Flag-TDP-43-12xQ/N F4L before (−DOX) and after (+DOX) induction of TDP-43 aggregation. Relative expression of proteins was quantified with western blot (**c**). Fibrillarin (FBL) and GAPDH were used as loading controls for nuclear and cytoplasmic fractions. Unpaired Student’s t-test was used to determine significant differences between samples (significance levels: *for p < 0.05, ***for p < 0.001). Error bars represent s.e.m.

In HEK293 Flp-in Flag-TDP-43-12x-Q/N F4L cells (referred to as HEK TDP-12xQ/N-F4L hereafter) TDP-43 aggregation was induced by addition of doxycycline (DOX) to the growth medium (Fig. 1). The aggregates were observed in the nucleus and the cytoplasm and could be detected both by anti-TDP-43 and anti-Flag antibodies. After 72 hours, the nuclear levels of soluble TDP-43 significantly dropped to 37.4 ± 4.7% (mean ± s.e.m., n = 3) as determined by western blot (Fig. 2a,c).

Splicing of more than 1000 mRNAs is affected by decreased cellular levels of TDP-43^2^,^26^. It has been previously shown that *POLDIP3 (SKAR)* and *RANBP1* splicing is under control of TDP-43^2^,^26^,^32^. TDP-43 knockdown or sequestration promoted splicing of *POLDIP3* (SKAR) exon 3 and inclusion of *RANBP1* exon 5 in both SH-SY5Y and HEK TDP-12xQ/N-F4L in the same pattern (Fig. S1), thus validating their applicability for this study.

In HEK TDP-12xQ/N-F4L western blot analyses showed differential expression of DNMT3A, HNRNPA3, EIF4A3, POLDIP3, PABPC1, and RANBP1, before and after induction of TDP-43 aggregation, which correlated with proteomic results of TDP-43 knockdown in SH-SY5Y (Table 1, Fig. 2, and Table S2). Namely, nuclear expression of DNMT3A, HNRNPA3, and PABPC1 dropped, whereas expression levels of POLDIPβ and EIF4A3 increased and cytoplasmic levels of RANBP1 dropped.

**Figure 3:**
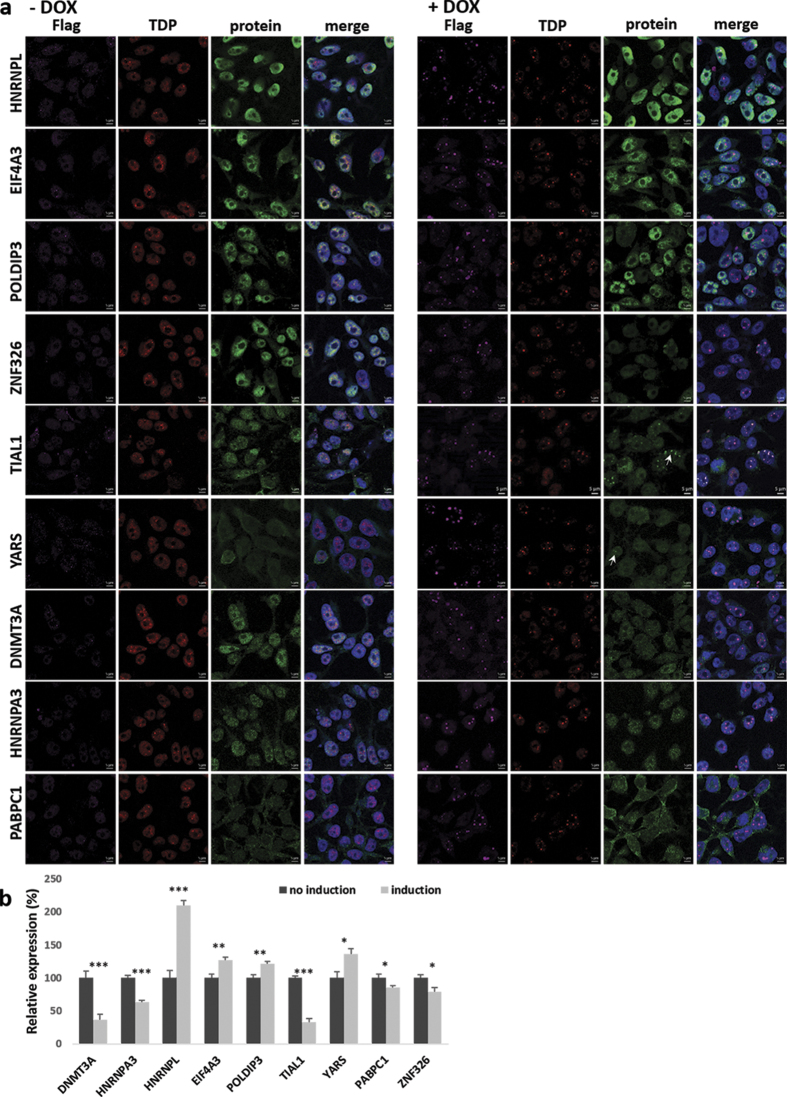
Expression of five nuclear proteins and one predominantly cytoplasmic protein (YARS) increased, and of DNMT3A, HNRNPA3, and PABPC1 dropped in the nuclear fraction of HEK Flp-in Flag-TDP-43-12xQ/N F4L after induction of TDP-43 aggregation. (**a**) Non-induced cells (−DOX) and cells after induction of TDP-43 aggregation (+DOX). Arrows mark protein aggregates, which in the case of TIAL1 colocalise with TDP-43 aggregates. Scale bars are 5 μm. (**b**) Immunofluorescence signal intensity quantification. Unpaired Student’s t-test was used to determine significant differences between samples (significance levels: *for p < 0.05, **for p < 0.01, ***for p < 0.001). Error bars represent s.e.m.

**Figure 4:**
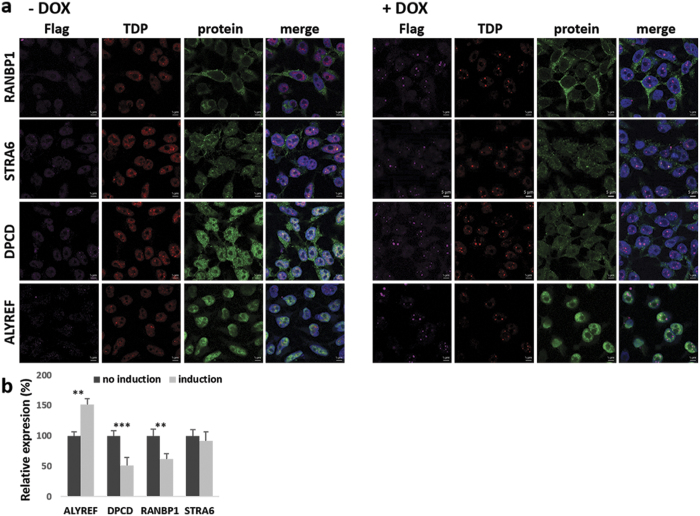
Cytoplasmic expression of RANBP1, STRA6, and DPCD dropped, while expression of, predominantly nuclear protein, ALYREF, increased in HEK Flp-in Flag-TDP-43-12xQ/N F4L after induction of TDP-43 aggregation. (**a**) Non-induced cells (−DOX) and cells after induction of TDP-43 aggregation (+DOX). Scale bars are 5 μm. (**b**) Immunofluorescence signal intensity quantification. Unpaired Student’s t-test was used to determine significant differences between samples (significance levels: **for p < 0.01, ***for p < 0.001). Error bars represent s.e.m.

Next we quantified the expression changes of the selected proteins following immunofluorescence (IF) staining (Figs 3 and 4). Signal intensity quantifications showed that 12 out of 13 proteins had statistically significant expression level changes after induction of aggregation (Figs 3b and 4b, Table S3). Most importantly, for 10 out of 13 proteins, the change in expression levels correlated with knockdown data from Štalekar *et al.*^27^. After TDP-43 aggregation, relative levels of nuclear proteins HNRNPL, EIF4A3, POLDIP3, and YARS increased, whereas nuclear levels of DNMT3A, HNRNPA3, and PABPC1 dropped (Fig. 3b). Although ALYREF is localised mainly in the nucleus, we observed its upregulation in the cytoplasmic fraction of HEK TDP-12xQ/N-F4L after TDP-43 aggregation (Fig. 4). We have previously shown reduced levels of RANBP1 after TDP-43 silencing in SH-SY5Y^27^ and herein confirmed this occurrence also in HEK TDP-12xQ/N-F4L after induction of TDP-43 aggregation (Figs 2b,c and 4). Finally, also DPCD cytoplasmic levels dropped in HEK TDP-12xQ/N-F4L after TDP-43 aggregation (Fig. 4).

In addition, some discrepancies between the proteomic data obtained for HEK TDP-12xQ/N-F4L in comparison to RNAi in SH-SY5Y may be on account of the difference in experimental model used (knockdown vs. aggregation), the cell line (HEK vs. SH-SY5Y), the experimental conditions (induction vs. transfection, media), which in turn reflect a moderate difference in the regulation of expression of proteins in question. There is also some variation to be expected from the antibodies used, as some perform better in denaturing conditions of western blot, while others recognise better the proteins in cells (IF). The recognition of the latter can also be influenced by method of fixation of cells (for instance using paraformaldehyde or methanol) or the accessibility of the epitope against which the antibody was raised due to specific protein conformation.

## Discussion

In this study, using a cellular model of TDP-43 sequestration, we have examined expression variations of a series of proteins previously shown to be differentially expressed following TDP-43 knockdown^27^. As has been previously demonstrated in HEK293 Flp-in Flag-TDP-43-12x-Q/N F4L cell line, loss of TDP-43 function was achieved because endogenous TDP-43 was able to interact fully with Flag-TDP-43-12xQ/N aggregates and ended up sequestered in both nuclear and cytoplasmic insoluble aggregates^30^. The aggregation was enforced by a Q/N-rich region of TDP-43, which itself is involved in aggregate formation and in the interaction of TDP-43 with inclusions^4^. Thus, cells had drastically reduced levels of active nuclear TDP-43 and presented a suitable model to test the impact of TDP-43 sequestration on expression and redistribution of a selection of proteins, whose functions have already been shown to be altered by TDP-43 knockdown^27^. As TDP-43 inclusions in HEK TDP-12xQ/N-F4L formed in the nucleus as well as the cytoplasm, it is not clear whether the change in expression of TDP-43 and other proteins was due to the GOF or LOF mechanisms, although our study suggests that the aggregation of TDP-43 causes proteomic changes in the cells akin to TDP-43 LOF.

TDP-43 is an RNA/DNA binding protein with multiple functions. For this reason, its downregulation influences a large number of RNA and protein targets. Decreased TDP-43 cellular levels affected splicing of 158 exons in neuroblastoma cells and altered 965 splicing events in adult mouse brain^2^,^26^,^30^. A remarkable alteration of splicing of polymerase delta-interacting protein 3 (*POLDIP3/SKAR*) has been previously noted as a result of the depletion of TDP-43^2^,^26^,^32^,^33^. The decreased inclusion of exon 3 in *POLDIP3* gene has been reported to favour the synthesis of the β isoform in respect to the main α isoform^32^,^33^,^34^. After TDP-43 sequestration we confirmed this splicing pattern, in both SH-SY5Y and HEK TDP-12xQ/N-F4L, detecting higher levels of β-isoform at the mRNA level^30^, and this observation was mirrored at the protein level. The increased expression of the shorter *POLDIP3* mRNA variant was also observed in ALS patients’ spinal motor neurons^32^. In this study, we have employed an antibody that recognises both α and β isoforms of POLDIP 3. After densitometric quantification of western blot data we confirmed the upregulation of β isoform, 252.0 ± 6.2% (mean ± s.e.m., n = 3), and the concomitant downregulation of α isoform, 13.3 ± 17.6% (mean ± s.e.m., n = 3). However, immunofluorescence showed only a smaller increase, 121.6 ± 4.0% (mean ± s.e.m., n = 3), as it was a cumulative measurement of α and β POLDIP3 isoform.

Our comparative analysis of siRNA-mediated knockdown versus sequestration and aggregation of TDP-43 has allowed us to confirm two possible connections between loss of TDP-43 functional activity and disease. First of all, our analyses add further support to the possible link between TDP-43 and nonsense-mediated decay. In fact, POLDIP3 was also reported to associate with the exon junction complex (EJC), which is recruited to exon junctions during splicing^35^. Furthermore, after TDP-43 aggregation, one of the core proteins of EJC, eukaryotic translation initiation factor 4A3 (EIF4A3), was also upregulated in the nuclear fraction of HEK TDP-12xQ/N-F4L. In addition, EIF4A3 is connected to ALYREF, which is known to recruit export factors during the formation of export competent messenger ribonucleoprotein complexes (mRNPs), thus enabling mRNA export^36^. Although POLDIP3 and EIF4A3 were upregulated in the nucleus, suggestive of stalled mRNA export and protein production, ALYREF was upregulated in the cytoplasm. ALYREF shuttles between nucleus and cytoplasm and beside mRNA export it has been implicated in linking splicing with transcription^37^,^38^. This suggests that TDP-43 aggregation induces staling of ALYREF in the cytoplasm, compromising transcription, nuclear RNA stability, and mRNA export.

Secondly, in a *Drosophila* study, ALYREF was singled out as potential modifier of G_4_C_2_ expansion related toxicity^39^. (G_4_C_2_)_n_ hexanucleotide repeat expansion mutation in the *C9orf72* gene, which can span from several hundred to several thousand repeats, is the major genetic cause of ALS and FTLD leading to TDP-43 proteinopathy^40^,^41^,^42^. As toxic G_4_C_2_ RNA are sequestered in nuclear foci, ALYREF may act as part of a control mechanism that retains unspliced or faulty RNAs in the nucleus^36^. The question arises weather the redistribution of ALYREF to the cytosol, as a consequence of TDP-43 sequestration, might therefore be a mechanism of inhibiting nuclear transport due to aggregation induced proteotoxicity of TDP-43.

Thirdly, TDP-43 sequestration impacts several HNRNP proteins by either increasing their concentration in the nucleus, or by nuclear clearance, thus altering RNA metabolism and potentially leading to ALS and FTLD. Following TDP-43 aggregation we observed altered levels of HNRNPA3 and HNRNPL. Previously, HNRNPA3 was found to be aggregated in cytosolic TDP-43 negative inclusions in the brains of patients with *C9orf72* expanded repeats^43^. In FTLD-TDP patients, also increased expression of HNRNPA1/A2 was detected^44^. On the other hand, in ALS patient motor neurons loss of HNRNPA1 expression was concomitant with TDP-43 cytoplasmic inclusions^45^. The expression of HNRNP proteins is probably regulated not only by sequestration of TDP-43 but by network of several other RNA-binding proteins (RBP), whose expression might also depend on the cell/tissue type tested or the course of disease progression. In addition to HNRNP proteins, PABPC1 is another RBP whose expression was observed to be altered after TDP-43 aggregation. Namely, its nuclear expression dropped in HEK TDP-12xQ/N-F4L. PABPC1 is predominantly cytoplasmic protein that shuttles between the nucleus and the cytoplasm. Together with TDP-43 it accumulates in the stress granules leading to translational repression^46^,^47^. Its mislocalisation in robust cytoplasmic inclusions has been observed in ALS spinal cord motor neurons^48^. We did not observe aggregation of PABPC1 in HEK TDP-12xQ/N-F4L but detected colocalization of TDP-43 aggregates with another stress granule marker, TIAL1. After TDP-43 knockdown in SH-SY5Y TIAL1 nuclear expression increased, however, we have not observed this when quantifying fluorescence levels in HEK TDP-12xQ/N-F4L. This might be specific to the aggregates formed or the antibody used as it detects both TIAL1 and TIA1, thus, we were most likely quantifying fluorescence signals for both proteins.

Finally, we have previously shown in SH-SY5Y that depletion of TDP-43 influences intracellular transport through downregulation of RANBP1 and herein confirmed the expression drop in HEK TDP-12xQ/N-F4L model^27^. In addition, in both cell lines we confirmed that silencing/aggregation of TDP-43 enables inclusion of exon 5 in *RANBP1* transcript, in turn decreasing the level of protein.

In conclusion, although the pathological relevance of these processes *in vivo* still needs to be determined, our cellular analysis has added further support that aggregation and sequestration model overlaps TDP-43 LOF following its knockdown by siRNA. As a consequence, the major hits from this comparison might represent good candidates to be followed up in further studies that aim to elucidate the causes of ALS/FTLD pathology.

## Methods

### Cell culture

HEK293 Flp-in Flag-TDP-43-12x-Q/N F4L cell line was established as previously described^30^. Cells were grown in DMEM-Glutamax-I (Gibco) supplemented with 10% tet-free fetal bovine serum (Biowest), 100 U/ml penicillin-streptomycin (Gibco), 100 μg/ml hygromycin B (Sigma) and 10 μg/ml blastycydin (Sigma). The induction of expression of Flag-TDP-43-12x-Q/N proteins was achieved by adding 1 μg/ml doxycycline (Sigma) to the culture medium for 72 hours.

### Cell fractionation

Cells were grown in 6-well plates and were harvested in cold CLB buffer (50 mM Tris, pH 7.4, 10 mM NaCl, 0.5% Igepal Ca-630 (Sigma-Aldrich), 0.25% Triton X-100, cocktail of protease inhibitors (Roche)) and centrifuged for 5 min at 3000 g at 4 °C. Supernatants were transferred to fresh tubes and re-centrifuged at 16100 g at 4 °C for 10 min. Obtained supernatants were used as cytoplasmic fractions. The first pellets were washed three times in cold CLB, resuspended in 1X SDS loading buffer without bromophenol blue, sonicated, boiled for 5 min and re-centrifuged. Resulting supernatants were saved as nuclear fractions. The protein concentration in the fractions were determined by Bio-Rad DC Protein Assay.

### Western blot

Reducing SDS-PAGE was run on 4–12% SDS precast gels (C.B.S. Scientific) loaded with 10–20 μg of protein samples in 1X SDS loading buffer with 100 mM dithiothreitol at 175 V. Wet transfer onto nitrocellulose membrane (GE Healthcare) was carried out at 200 mA for 90 min. Membranes were blocked with 5% non-fat dry milk in TBS with 0, 05% Tween-20 (TBST, Sigma) at room temperature for 1 hour. Primary antibodies (Table S1) diluted in blocking medium were incubated over night at 4 °C with gentle rocking. Membranes were washed three times with TBST and incubated in the dark with fluorescently-labelled secondary antibodies (Goat anti-Rabbit IgG (H + L) Cross Adsorbed Secondary Antibody, Dylight 650 conjugate and Goat anti-Mouse IgG (H + L) Cross Adsorbed Secondary Antibody, Dylight 550 conjugate, both Thermo Fischer Scientific) at room temperature for 1 hour. After three washes with TBST, images were acquired using GelDoc System (Bio-Rad). ImageLab software (Bio-Rad) was used for densitometric analysis. Each experiment was performed three times in triplicates (n = 3). Additionally, samples were loaded in triplicates. Loading controls were run on the same blot for each experiment.

### Immunofluorescence

Cells were grown on poly-L-lysine (Sigma) coated glass coverslips and were washed once with PBS and fixed with 4% paraformaldehyde in PBS for 15 min. Then they were permeabilised with 0.1% Trition X-100 (Sigma) in PBS for 5 min. Blocking was performed with 3% BSA (Sigma) in PBS for 1 hour. Cells were incubated with primary antibodies (Table S1) diluted in blocking solution for 1 hour at room temperature, followed by washing with PBS and incubation in the dark with fluorescently-labelled secondary antibodies (Donkey Anti-Rabbit IgG (H + L) Alexa Fluor 488, Goat anti-Mouse IgG (H + L) Alexa Fluor 633, Goat Anti-Rat IgG (H + L) Alexa Fluor 555, all Invitrogen) for 1 hour. After washing with PBS cells were stained with DAPI (Sigma) for 7 min and washed with PBS. Coverslips were mounted using FluorSave reagent (Millipore). Images were acquired with Zeiss LSM 710 inverted confocal laser scanning microscope with a Plan-Apochromat 63 ×/1.4NA M27 oil immersion objective using immersion oil (Carl Zeiss). DAPI, Alexa Fluor 488, Alexa Fluor 555, and Alexa Fluor 633 were excited at 405, 488, 543 or 633 nm, respectively. The zoom factor was set to 1 × . X- and Y-scanning sizes were each 1024 pixels. All images were further cropped in ZEN 2010 B SP1 software and scale bars were added.

ImageJ was used to quantify immunofluorescence signal intensities as follows. Several regions of interest (ROI) were selected in the appropriate cellular location, and the mean fluorescence of detected protein was measured, along with several adjacent background readings. The total corrected cellular fluorescence, (CTCF = integrated density−(area of selected cell × mean fluorescence of background readings), was calculated for each ROI. The averages and s.e.m. were determined for several images for each marker in induced cells compared to non-induced, where expression was set to 100%. Statistical analysis (2-sided unpaired Student’s t-tests) was performed.

### Statistical analysis

Quantitative values of protein bands in western blots were normalized to GAPDH in cytoplasmic fractions and to fibrillarin (FBL) in nuclear fractions. Relative protein expression levels in TDP-43 aggregating HEK TDP-12xQ/N-F4L were calculated towards non-induced controls. For immunofluorescence quantification, immunofluorescence signal intensities of cells with induced TDP-43 aggregation and non-induced controls were compared and relative protein expression levels were calculated. Statistical significance of differential expression of proteins according to western blot and immunofluorescence was determined with unpaired Student’s t-test analysis. Student’s t-test was performed in Microsoft Excel Professional Plus 2013. A *p*-value of <0.05 was considered significant.

## Additional Information

**How to cite this article**: Prpar Mihevc S. *et al.* TDP-43 aggregation mirrors TDP-43 knockdown, affecting the expression levels of a common set of proteins. *Sci. Rep.* **6**, 33996; doi: 10.1038/srep33996 (2016).

## Supplementary Material

Supplementary Information

## Figures and Tables

**Table 1:** Selection of significantly changed proteins in the nuclear and cytoplasmic fractions of SH-SY5Y and corresponding expression data following TDP-43 knockdown.

HUGO symbol	Proteome				Transcriptome	
	Protein name	p-value*	KD/C ratio*	cell fraction	∆T**	∆T rank**
EIF4A3	Eukaryotic initiation factor 4A-3	0.0195	9.50	nuc	1.02	0
TARDBP	TAR DNA binding protein	0.0008	0.18	nuc	3.16	5.7
YARS	Tyrosine-tRNA synthetase	0.0046	5.38	nuc	1.07	0
DNMT3A	DNA methyltransferase 3α	0.0000	0.23	nuc	1.25	0.25
POLDIP3	DNA polymerase delta interacting protein 3	0.0009	3.10	nuc	0.95	0.01
HNRNPL	Heterogeneous nuclear ribonucleoprotein L	0.0016	2.82	nuc	1.04	0
ZNF326	Zinc finger protein 326	0.0166	2.53	nuc	1.04	0
PABPC1	poly(A) binding protein cytoplasmic 1	0.0018	0.40	nuc	/	/
HNRNPA3	Heterogeneous nuclear ribonucleoprotein A3	0.0075	0.40	nuc	1.09	0.03
TIAL1	TIA1 cytotoxic granule associated RNA binding protein like 1	0.0081	2.38	nuc	1.35	0.54
STRA6	Stimulated by retinoic acid 6	0.0001	0.08	cyt	1.58	1.31
DPCD	deleted in primary ciliary dyskinesia homolog	0.0007	0.16	cyt	0.94	0.02
ALYREF	Aly/REF export factor	0.0166	3.62	cyt	1.03	0
RANBP1	RAN binding protein 1	0.0040	0.44	cyt	1.38	0.5

P-value obtained from Fisher’s exact test was calculated using total spectrum counts of identified peptides from control and three TDP-43-knockdown replicates; p < 0.05 was regarded as statistically significant. KD/C ratio represents the protein ratio between TDP-43 knockdown (KD) and control (C). nuc–nuclear fraction, cyt–cytoplasmic fraction. ∆T-fold change in transcript abundance, ∆T rank-modified t-test to sort the genes based on ΔT significance^31^. ^*^Data from Štalekar *et al.*^27^; ^**^data from Tollervey *et al.*^2^.
