# Supplementary material for: TDP-43 aggregation mirrors TDP-43 knockdown, affecting the expression levels of a common set of proteins
Source: Sci Rep. 2016 Sep 26;6:33996. doi: 10.1038/srep33996 (PMC5036055; doi:10.1038/srep33996)
Supplement: Supplementary Information [file srep33996-s1.pdf]

Supplementary Information

TDP-43 aggregation mirrors TDP-43 knockdown, affecting the expression levels of a common set of proteins

Sonja Prpar Mihevc[1], Marco Baralle[2], Emanuele Buratti[2], and Boris Rogelj[1,3,4*]

[1] Department of Biotechnology, Jožef Stefan Institute, Jamova 39, SI-1000 Ljubljana, Slovenia

[2] ICGEB – International Centre for Genetic Engineering and Biotechnology, Padriciano 99, IT-34149 Trieste, Italy

[3] Biomedical Research Institute BRIS, Puhova 10, SI-1000 Ljubljana, Slovenia

[4] Faculty of Chemistry and Chemical Technology, University of Ljubljana, Večna pot 113, SI-1000 Ljubljana, Slovenia

* Corresponding author; email: boris.rogelj@ijs.si

Supplementary figure

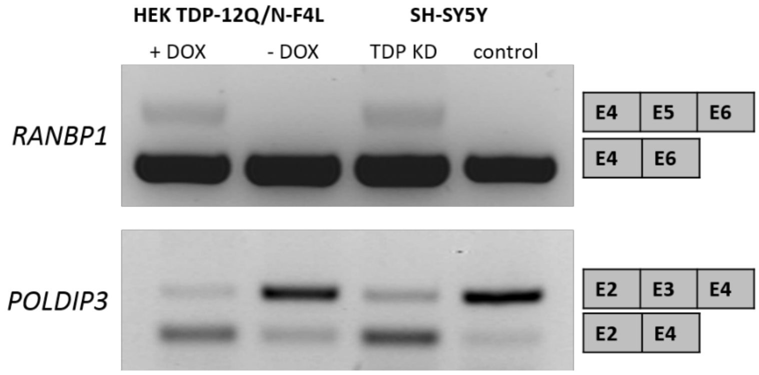

Supplementary Figure S1. Splicing pattern for *RANBP1* and *POLDIP3* is similar in HEK Flp-in Flag-TDP-43-12x-Q/N F4L and SH-SY5Y after and before TDP-43 aggregation/knockdown.

Supplementary tables

**Target/Antigen**	**Source**	**WB**	**IF**
STRA6	Proteintech, 22001-1-AP	1/100	1/50
DPCD	Proteintech, 20936-1-AP	1/100	1/50
ALYREF	Santa Cruz, sc-32311	1/50	1/100
RANBP1	Abcam, ab2937	1/2000	1/1000
RANBP1	Novus Biologicals, NB100-79814	1/1000	1/400
EIF4A3	Proteintech, 17504-1-AP	1/1000	1/50
YARS	Santa Cruz, sc-166741	1/30	1/50
DNMT3A	Santa Cruz, sc-20703	1/1500	1/100
DNMT3A	Novus Biologicals, NBP1-85961		1/200
POLDIP3	Proteintech, 17466-1-AP	1/500	1/50
HNRNPL	Santa Cruz, sc-32317	1/200	1/100
ZNF326	Proteintech, 25147-1-AP	1/500	1/50
PABPC1	Proteintech, 10970-1-AP	1/250	1/25
HNRNPA3	Santa Cruz, sc-133665	1/200	1/50
TIAL1	Santa Cruz, sc-28237	1/200	1/50
TDP-43	Millipore, MABN150		1/200
TDP-43	Proteintech, 10782-2-AP	1/5000	
Flag-tag	Proteintech, 60002-1-IG		1/50
Flag-tag	Proteintech, 20543-1-AP		1/50
GAPDH	Santa Cruz, sc-25778	1/1000	
GAPDH	Proteintech, 60004-1-ig	1/5000	
FBL	Santa Cruz, sc-25397	1/500	
FBL	Santa Cruz, sc-166001	1/250	

Supplementary Table S1. Antibodies used in this study. Dilutions for western blot (WB) and immunofluorescence (IF) are presented.

Protein	- DOX		+ DOX		p-value
	mean	s.e.m.	mean	s.e.m.	
**TDP-43**	100	8.88	37.44	4.71	0.03
**DNMT3A**	100	6.42	56.86	14.61	0.04
**HNRNPA3**	100	8.88	73.30	9.69	0.05
**HNRNPL**	100	6.70	103.99	4.16	0.62
**EIF4A3**	100	6.49	132.22	5.22	0.04
**POLDIP3α**	100	15.97	13.28	17.63	0.00
**POLDIP3β**	100	18.20	252.02	6.21	0.03
**TIAL1**	100	19.57	138.18	18.69	0.61
**YARS**	100	2.57	106.26	3.04	0.18
**PABPC1**	100	4.34	67.54	2.37	0.00
**ZNF326**	100	7.27	132.89	7.90	0.23
**ALYREF**	100	3.28	121.42	10.53	0.16
**DPCD**	100	4.68	92.35	6.34	0.34
**RANBP1**	100	2.74	56.18	3.92	0.03
**STRA6**	100	17.20	73.93	14.06	0.24

Supplementary Table S2. Relative expression of proteins (in %) quantified with western blot. Unpaired Student’s t-test was used to determine significant differences between samples (n =3). Calculated s.e.m. and p-values are provided.

Protein	- DOX		+ DOX		p-value
	mean	s.e.m.	mean	s.e.m.	
**DNMT3A**	100	10.27	36.90	7.86	0.0000
**HNRNPA3**	100	3.58	63.73	2.68	0.0000
**HNRNPL**	100	11.07	210.53	7.05	0.0000
**EIF4A3**	100	5.69	126.93	4.52	0.0017
**POLDIP3**	100	5.36	121.61	4.02	0.0047
**TIAL1**	100	2.72	33.39	5.12	0.0000
**YARS**	100	9.98	136.67	8.38	0.0226
**PABPC1**	100	6.02	85.99	2.39	0.0497
**ZNF326**	100	5.26	78.91	6.85	0.0121
**ALYREF**	100	6.91	151.40	9.98	0.0099
**DPCD**	100	8.11	50.93	13.42	0.0004
**RANBP1**	100	10.71	61.57	8.75	0.0055
**STRA6**	100	9.72	91.55	15.01	0.6269

Supplementary Table S3. Relative expression of proteins (in %) determined by immunofluorescence signal intensity quantification. Unpaired Student’s t-test was used to determine significant differences between samples (n = 50 – 150 cells). Calculated s.e.m. and p-values are provided.

Supplementary methods

RNA isolation and splicing analysis

SH-SY5Y cells were grown in DMEM/F12-Glutamax (Gibco) supplemented with 10% FBS (Gibco) and 100 U/ml penicillin-streptomycin. siRNA targeting TDP-43 was obtained from Invitrogen (Stealth) and was transfected by PepMute (SignaGen Laboratories), using 5 nM siRNA. For RNA isolation SH-SY5Y cells were harvested after 48 hours and HEK293 Flp-in Flag-TDP-43-12x-Q/N F4L cells 72 hours after induction of TDP-43 aggregation. Cell lines were overlaid with QIAzol reagent and scraped from the surface of the dishes. Total RNA was isolated from the aqueous phase, using miRNeasy mini kit (Qiagen) according to manufacturer's instructions. Reverse transcription-polymerase chain reaction (RT-PCR) was performed using High-Capacity cDNA Reverse Transcription Kit (ThermoFisher Scientific). PCR primer pairs used for splicing analysis were 5'-ACTGTTCCGATTTGCCTCTG-3', 5'-TGCATTCTCAGCATTCAGGA-3' for *RANBP1* and 5'-TGCTCTGAAGCTCACCAAAA-3', 5'-GGAACGGAAGCTATACCATCAT-3' for *POLDIP3*. Amplicons were visualized on 2% agarose gel.
